# Sex and gender differences in technology needs and preferences among informal caregivers of persons with dementia

**DOI:** 10.1186/s12877-020-01548-1

**Published:** 2020-05-18

**Authors:** Chen Xiong, Bing Ye, Alex Mihailidis, Jill I. Cameron, Arlene Astell, Emily Nalder, Angela Colantonio

**Affiliations:** 1 Rehabilitation Sciences Institute, Faculty of Medicine, University of Toronto, 500 University Avenue, Suite 160, Toronto, Ontario M5G 1V7 Canada; 2 Toronto Rehabilitation Institute-University Health Network, 550 University Ave, Toronto, M5G 2A2 Ontario Canada; 3 Acquired Brain Injury Research Lab, University of Toronto, 500 University Ave, Room 260, Toronto, M5G 1V7 Ontario Canada; 4 Department of Occupational Science & Occupational Therapy, University of Toronto, 500 University Avenue, Suite 160, Toronto, Ontario M5G 1V7 Canada; 5 Dalla Lana School of Public Health, University of Toronto, 155 College St, Room 500, Toronto, M5T 3M7 Ontario Canada

**Keywords:** Caregiving, Dementia, Sex and gender, Technology

## Abstract

**Background:**

Dementia is a major public health concern associated with significant caregiver demands and there are technologies available to assist with caregiving. However, there is a paucity of information on caregiver needs and preferences for these technologies, particularly from a sex and gender perspective. To address this gap in research, the objectives of this study are to examine (1) the knowledge of technology, (2) perceived usefulness of technology, (3) feature preferences when installing and using technology and (4) sex and gender influences on technology needs and preferences among family caregivers of persons with dementia (PWD) across North America.

**Methods:**

A secondary analysis was conducted on an existing cross-sectional survey with family caregivers of PWDs. Respondents were recruited through the Alzheimer Society of Canada, the Victorian Order of Nurses and Adult Day Programs and other Canadian health care provision institutes. Descriptive statistics, bivariate and multivariate analyses were used to describe the study sample, uncover differences between male and female caregivers and examine sex and gender influences on caregivers’ technology needs and preferences.

**Results:**

A total of 381 eligible responses were received over a nine month data collection period. The majority of respondents did not know much about and never used any technologies to assist with caregiving. “Being easy to install”, “easy to learn how to use” and “cost” were identified as the most important features when purchasing and setting up technology, while “reliability” was identified as the most important feature when using technology. Most respondents were willing to pay up to $500 to acquire individual technologies. Controlling for other socio-demographic variables, female respondents were more likely to have some or more knowledge about technology for caregiving while male respondents were more willing to pay higher amounts for these technologies compared to their female counterparts.

**Conclusions:**

As one of the first studies of its kind, our findings represent a step towards the incorporation of sex and gender considerations such as cost and reliability in technology design and promotion for caregivers. Future efforts are warranted to establish an in-depth understanding of sex and gender influences in relation to other social and environmental factors.

## Background

Dementia is a major public health concern worldwide. Across the globe, more than 50 million individuals are currently living with dementia and that number is expected to more than triple to over 152 million by 2050 [1]. At present, the total estimated worldwide societal cost of dementia is approximate US$1 trillion, a figure that will rise to US$2 trillion by 2030. Dementia is an overall term that describes a wide range of symptoms associated with a decline in mental ability, and results from several conditions, the most common being Alzheimer’s disease [2]. In addition to symptoms associated with cognitive decline, persons with dementia (PWD) also experience behavioural and psychological disturbances such as depressive mood, anxiety, restlessness, and agitation among others [3].

Age is the biggest risk factor for dementia, and the aging population means that an increasing number of family members are providing care for a PWD. In 2018, family caregivers provided more than 82 billion unpaid hours of care, a number that is expected to continue rising [4]. Caring for a family member with dementia can be a highly stressful experience for family caregivers and may contribute to a decline in their own mental health as well as increasing the risk of serious illness [5–8]. To improve the health and psychosocial outcomes of PWD and their caregivers, a range of technological interventions have been developed [9, 10]. These technologies include but are not limited to telehealth and web-based support programs, fall alarms, Global Positioning System (GPS) tracking devices, home monitoring cameras and devices to switch off stoves and water [11]. While some of these technologies can reduce caregiving burden and diminish some of the physical and emotional effort entailed in supporting family caregivers, there remain a number of significant challenges and barriers with respect to the use and adoption of these technologies. Specifically, technologies have been perceived as being too complex and lack explicit ethical values and considerations [12–15]. Additionally, systemic shortcomings such as a lack of awareness, accessibility and integration with current infrastructure have limited the ability for these technologies to adequately address the needs of caregivers [16]. Given these barriers and importance of understanding the needs of caregivers during the technology development process, this study seeks to bridge this gap by understanding the current use, awareness, needs and preferences of these technologies among caregivers.

To date, several models and frameworks have been developed to conceptualize the factors that influence technology acceptance and adoption, most notably the Technology Acceptance Model (TAM) [17–19]. Adapted from the theory of reasoned action [20], the TAM was developed to address why users accept or reject particular technologies [21]. As part of the model, external variables influence the technology’s perceived usefulness and ease of use, which in turn will affect the attitudes towards and behavioural intention to use the technology [21]. Given the widespread acceptance of the TAM in conceptualizing technology use, it was adopted in this study as the theoretical framework that shaped the analyses.

Within the context of the current study, sex refers to “… the biological and physiological characteristics that distinguish males from females” [22]. Gender refers to “… socially constructed roles, relationships, behaviours, relative power, and other traits that societies ascribe to women and men” [22]. While these constructs are distinct, we recognize that they are interrelated and, on a continuum. As such, it is important to take into account both constructs in the analyses and we will be referring to them collectively as ‘sex and gender’ for the remainder of this paper. Among the general population, sex and gender have played a significant role in determining the intention of accepting new technology. Additionally, men were more adept at using technology, specifically devices such as computers, email services and electronic data managements [23]. While there has been much study with respect to the sex and gender gap in general technologies, there remains a lack of research on technology perceptions among informal caregivers despite considerable sex and gender differences with respect to well-being, psychosocial and overall health [24–27]. Specifically, female caregivers report higher levels of depressive symptomatology and are at a higher risk for clinical depression compared to their male counterparts [26]. In line with this, female caregivers are found to report poorer physical health and more emotional distress due to caregiving compared to their male counterparts [28–30]. More recently, a systematic search of the literature on caregiving technology revealed few studies [31, 32] that have assessed informal caregiver needs with respect to technology from a sex and gender lens. Specifically, there was a lack of overall awareness of caregiving technology among family caregivers of PWD, with female Chinese caregivers of PWD significantly more receptive towards technology compared to their male counterparts [31]. Similarly, female caregivers of PWD were more appreciative of the use of tracking devices to monitor care recipient whereabouts compared to males [32]. While both studies highlighted important differences in the preferences and reception of technology among male and female caregivers of PWD, the studies were either based on a small sample size [31] or outside of North America [32]. Given the lack of attention to sex and gender within this field of caregiving and technology as well as across research, a number of governmental organizations including the European Commission and Canadian government have identified sex and gender as priority areas of research as well as policy initiatives [33, 34].

To address this gap in research and priority area, the objectives of this study are to examine

1. The knowledge and use of technology to support caregiving
2. Perceived usefulness of technology
3. Feature preferences when installing and using technology
4. Sex and gender influences on technology needs and preferences among family caregivers of PWD.

Based on the findings from the limited existing literature, we hypothesized that few caregivers of PWD currently use any technologies for caregiving and most have little to no knowledge of these technologies. Given the paucity of literature in this area, we conducted an exploratory analysis to identify specific areas of caregiving where technology would greatly assist in and the specific features of technology valued by caregivers from a sex and gender perspective. Finally, in line with the results from a pilot study conducted by our team previously [31], we predicted that female caregivers will be more receptive towards technology for caregiving compared to their male counterparts.

## Methods

### Study population and design

This study was a secondary analysis of a previously administered cross-sectional survey that aimed to identify (1) the social factors that best explain the technology needs of PWD, (2) the needs that family caregivers of PWD have for technology that support cognition and activities of daily living (ADL), (3) the features and functions that would increase the likelihood of technology use, and (4) the criteria for creating a preliminary design framework [35]. Respondents were family caregivers of PWDs residing in North America. Respondents were recruited through the Alzheimer Society of Canada, including 52 of its chapters across Canada, as well as the Victorian Order of Nurses and Adult Day Programs and other Canadian health care provision institutes (subsequently referred to as ‘partner organizations’). Eligible respondents included those that met the following inclusion criteria (1) currently the primary informal caregiver (defined as any person providing care without financial compensation) of a PWD and (2) can speak, read and/or write in English. Respondents who were not the primary informal caregiver, had missing socio-demographic characteristics and those that were unable to complete the questionnaire due to language and/or communication barriers were excluded from the retrospective analysis.

Over a nine-month period of data collection, a total of 433 informal caregivers participated in the study. Of these 52 had missing socio-demographic information and were excluded, leaving 381 that were included in the data analyses. Comparisons between included and excluded respondents did not yield any significant differences in the socio-demographic characteristics of either the caregivers or the care recipients.

### Sampling procedures

Recruitment of respondents and data collection involved advertisements through newsletters, social media and flyers distributed by partner organizations between March 2013 and December 2013. Potential participants were given the option of completing the questionnaire electronically (i.e., respondents were provided with a link to an online survey hosted by LimeSurvey); by paper (i.e., hard copies mailed to the potential respondent with a return envelope and postage or distributed in person at information sessions at our partner organizations); in person (i.e., sit down sessions with a respondent) or over the phone where respondents were provided with a toll-free number to call and complete the questionnaire. Regardless of the method of participation, informed consent was collected from respondents. Specifically, informed consent was required for online respondents in order to continue with the survey, returned by mail together with the completed questionnaire, or collected in person or over the phone before completion of the questionnaire. Research Ethics Board (REB) approval was obtained from the University Health Network, Toronto, Ontario, Canada (REB #12–044).

### Measure

A questionnaire was designed to collect data on: (1) the social factors relating to caregiver’s technology needs and preferences, (2) technology needs of family caregivers of PWD, (3) features and functions that increase the likelihood of technology usage. Specifically, items in the questionnaire included in the analyses are caregiver and care recipient demographic information, scales to measure the abilities of PWD in completing ADL, family finances, caregiver knowledge and attitudes towards technology as well as features and functions of technology. Within the context of the questionnaire, technology is termed as ‘intelligent assistive technology’ and defined as any computer-based technologies designed to help individuals carry out their ADL and support individuals with cognitive impairment. Prior to administering the questionnaire, it was pilot tested among academics, professionals and experts in the field to ensure its validity and reliability and to determine the time needed to complete the questionnaire (approximately 30 min).

#### Independent variables

Caregiver socio-demographic variables were collected to describe the study population. These included age, sex and gender, marital status, race/ethnicity, rurality, education level, employment status, income level, housing arrangement, length of care and caregiving relationship. In addition, care recipient demographic variables such as care recipient age and ADL were also collected. As the main independent variable, information on respondents’ sex and gender was gathered through a multiple-choice item, with the question being ‘What is your gender?” and responses being ‘male’ or ‘female’.

#### Dependent variables

To assess objective (1), technology knowledge and current level of use, respondents were asked if they ever used technology to help with caregiving and rate their level of knowledge about the technologies available to support care of PWD. In line with the TAM, Objective (2), perceived usefulness and benefits of technology, were examined by gathering the perceived ability of technology to assist in care and allow the care recipient to remain at home. Specifically, respondents were provided with a list of ADL [36] and asked the extent technology would assist the care recipient with each of the activities. Objective (3), feature preferences of technology, were assessed by asking respondents to rank the features when installing and using technology from the most to least important. In addition, respondents were asked to indicate the amount that they were willing to pay to acquire technologies for caregiving. A full list of the questionnaire items used in the analyses is available in Supplementary Table 1.

### Data analysis

Statistical analyses were performed using SAS v. 9.3 (SAS Institute, Cary, NC). Descriptive statistics in the form of frequency distributions, percentages, means, standard deviations, and medians were used to examine the knowledge, perceived usefulness and feature preferences when installing and using technology for caregiving. Additionally, bivariate analyses involving t-tests, chi-square tests and Fisher exact tests were conducted to examine differences in socio-demographic variables between male and female respondents. To examine sex and gender influences on caregivers’ perceived usefulness, knowledge, use, and feature preferences of technology, multivariate analyses were conducted. Specifically, all caregiver and care recipient socio-demographic variables listed above were included in stepwise linear (to examine perceived usefulness of technology, a continuous variable), logistic (to examine technology use and knowledge, both nominal variables) and multinomial (to examine feature preferences of technology, an ordinal variable) regressions with an inclusion and retention cut-off *p*-values of 0.3 and 0.05 respectively. The order of variable insertion was determined using p-value selection, where the variable with the lowest p-value was added to the model first. As the main independent variable of interest, sex and gender was forced to be included in each of the models. A significance level of 0.05 was used for each regression analysis. Assumptions for each of the regression analyses were tested and satisfied.

## Results

Table 1 contains the overall as well as the sex and gender stratified socio-demographic characteristics for respondents included in the study and their care recipients. Approximately 79.8% of respondents were female. The mean age was 62.6 years (standard deviation (SD) = 12.7) and the median age was 63 years. The youngest was 20 and the oldest was 94 years of age. The majority of respondents were married, and more than half were spousal caregivers. With respect to the living location, 17.5% of the respondents were living in rural areas as identified by their postal codes. Almost all of the respondents identified themselves as White (91.6%). Most had a high school diploma or higher level of education (91.8%) and were either unemployed or retired (66.3%). With respect to family finances, 55.9% indicated that they either had some money left over or more than enough every month, while the remaining respondents indicated that they did not have enough or just had enough to make ends meet. More than half of the respondents lived in a single detached house (66.4%). On average, respondents spent 69.8 h (SD = 59.6) per week taking care of their care recipient, who had a mean age of 78.6 years (SD = 10.2). With respect to the length of care, a third of the respondents reported to having taken care of their care recipient for six or more years, 39.5% had taken care of their care recipient for three to five years and 28.7% have taken care of their care recipient for less than two years. Bivariate analyses examining sex and gender differences in sociodemographic variables found male caregivers to be significantly older than their female counterparts (*p* < 0.0001). In addition, a significantly greater proportion of female respondents were employed compared to males (*p* < 0.05). Significant relationships were also found between caregiver relationships and sex and gender (p < 0.05). Specifically, a greater proportion of male respondents were spousal caregivers compared to female respondents. No significant differences in the care recipient’s age and ADL score were found between male and female caregivers.

**Table 1:** Socio-demographic characteristics of respondents

Personal Characteristics	Overall	Male	Female	*p*-value
Age (mean years, SD)	62.6, 12.7	69.5, 12.8	60.8, 12.1	**< 0.0001**
Care recipient’s age (mean years, SD)	78.6, 10.2	76.7, 9.9	79.2, 10.3	0.0587
Care recipient’s activities of daily living score (mean, SD)	2.35, 0.69	2.39, 0.59	2.34, 0.71	0.583
	N (%)	n (%)	n (%)	
Marital Status				0.266
Married	265 (69.6)	62 (80.5)	203 (66.8)	
Race/Ethnicity				0.303
White	349 (91.6)	75 (97.4)	274 (90.1)	
Rural	64 (17.5)	14 (18.4)	50 (17.3)	0.866
Highest Level of Education				0.437
Less than a high school diploma	31 (8.1)	10 (12.9)	21 (6.9)	
High school diploma or equivalent	107 (28.1)	18 (23.4)	89 (29.3)	
College diploma	119 (31.2)	25 (32.5)	94 (31)	
University Degree	82 (21.5)	15 (19.5)	67 (22)	
Post graduate degree	42 (11)	9 (11.7)	33 (10.9)	
Employment Status				**0.0155**
Employed	128 (33.7)	17 (22.1)	111 (36.6)	
Unemployed	252 (66.3)	60 (77.9)	192 (63.4)	
Household income before taxes				0.121
Less than 25,000	66 (17.3)	10 (13)	56 (18.4)	
25,001–45,000	90 (23.6)	25 (32.5)	65 (21.4)	
45,001–65,000	81 (21.3)	12 (15.6)	69 (22.7)	
65,001–85,000	44 (11.6)	6 (7.8)	38 (12.5)	
85,001–100,000	51 (13.4)	10 (13)	41 (13.5)	
Over 100,000	49 (12.9)	14 (18.2)	35 (11.5)	
In general, how do your family finances work out?				0.521
Not enough/Just enough	168 (44.1)	31 (40.3)	137 (45.1)	
Some money left over/More than enough	213 (55.9)	46 (59.7)	167 (54.9)	
In what type of dwelling are you in?				0.789
Single detached house	253 (66.4)	50 (64.5)	203 (66.8)	
Other	128 (33.6)	27 (35.1)	101 (33.2)	
How long have you been caring?				0.339
Less than 1 years	26 (6.9)	3 (3.9)	23 (7.7)	
1 to 2 years	82 (21.8)	19 (24.7)	63 (21)	
3 to 5 years	149 (39.5)	35 (45.5)	114 (38)	
6 years or more	120 (31.8)	20 (26)	100 (33.3)	
Caregiving Relationship				**0.0134**
Spousal	182 (53.4)	53 (71.6)	129 (48.3)	
Children	128 (37.5)	18 (24.3)	110 (41.2)	

**Fig. 1:**
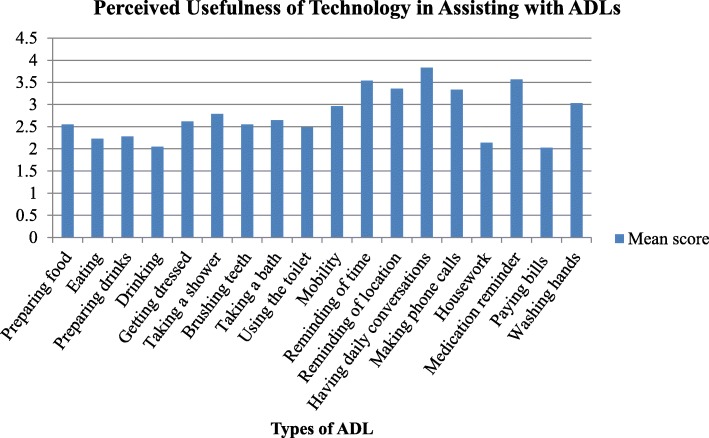
Perceived usefulness of technology in assisting with ADLs

In line with the study’s objectives, most of the respondents (83.7%) had none to little knowledge about these technologies. In addition, most (94.6%) had never used any technologies to help with their caregiving duties. Figure 1 presents the perceived usefulness of technology from a scale of 1 (not useful at all) to 6 (very useful), where a mean score of > 3.5 equates to ‘useful’. Among the respondents, technology was assessed as useful to assist with only three ADL: (1) having daily conversations with the care recipient (3.82, SD = 1.97), (2) reminding care recipients to take their medication (3.56, SD = 2.19) and (3) reminding care recipients of the current time (3.54, SD = 1.88). Technology was not perceived to be useful to assist with most other ADL including paying bills (2.02, SD = 1.73), drinking (2.05, SD = 1.51) and eating (2.22, SD = 1.57).

With respect to the feature preferences of technology, ‘easy to install’, ‘easy to learn how to use’ and ‘cost’ were each identified by approximately 30% of respondents as the most important potential feature when first setting up the technology (Table 2). When using technology, more than half identified ‘reliability’ as the most important potential feature, followed by 21.7% who indicated the ‘ability for the system to work without manual user input’ and 11.9% of respondents who indicated the ‘ease of getting help’ as the most important potential feature. With respect to how much respondents were willing to pay for technologies to assist with caregiving, 36.8% would pay less than $100, 43.0% were willing to pay between $101 and $500, 13.9% were willing to pay between $501 and $1000 and 6.3% were willing to pay more than $1000. No significant sex and gender differences were found with respect to these technology preferences.

**Table 2:** Feature preferences of technology among respondents

Feature preferences of Technology	Overall n (%)	Male n (%)	Female n (%)	*p*-value
Most important feature when setting up tech?	294 (100)			0.175
Easy to install	88 (29.9)	18 (27.7)	70 (30.6)	
Easy to learn how to use	85 (28.9)	27 (41.5)	58 (25.3)	
Cost	88 (29.9)	13 (20.0)	75 (32.6)	
Others^a^	33 (11.2)	7 (10.8)	26 (11.4)	
Most important feature of using tech?	286 (100)			0.0616
Easy to get help when broken	34 (11.9)	7 (11.1)	27 (12.1)	
Reliability	151 (52.8)	34 (54.0)	117 (52.5)	
Ability to work without manual user input	62 (21.7)	9 (14.3)	53 (23.8)	
Others^b^	39 (13.6)	13 (20.6)	26 (11.7)	

^a^Due to small cell sizes, response options including ‘Clear operating instructions’, ‘Availability of training’ and ‘Aesthetics of the technology’ were combined and categorized as ‘Others’

^b^Due to small cell sizes, response options including ‘Ability to set-up features on the device and customize its operation’, ‘Ability to receive performance reports about user performance and the system operation’ and ‘Accessible outside of the home (e.g. via Internet, smart phone, etc.)’ were combined and categorized as ‘Others’

A stepwise linear regression (Table 3) was conducted to examine the relationship between caregiver sex and gender and the perceived usefulness of technology. Controlling for socio-demographic variables listed in Table 1, sex and gender was not significantly associated with perceived usefulness of technology. Caregiver age was then added and found to be significantly associated with perceived usefulness of technology (*β* = − 0.015, *p* = 0.0089, 95% CI = − 0.028, − 0.0040). Specifically, older respondents were less likely to perceive technology as useful in assisting with caregiving activities (Table 3). The stepwise regression found no additional variables to be significantly associated with perceived technology usefulness.

**Table 3:** Results of stepwise linear regression analysis: perceived usefulness of technology (*n* = 293)

Variable	β (95% CI)	*p*-value
Sex and gender (ref: male)	0.16 (−0.17–0.49)	0.099
Caregiver age	−0.015 (−0.028 - -0.0040)	0.0089

Other socio-demographic variables such as marital status, ethnicity, education level, rurality, employment status, housing status, family finances, length of care, caregiving relationship, age of care recipient and care recipient’s ADL were not selected for entry into the stepwise regression

Stepwise logistic regression models were generated to examine the association between respondent’s sex and gender and technology knowledge (Table 4) as well as use (Table 5). Controlling for other socio-demographic variables listed in Table 1, only care recipient age was added and found to be significantly associated with the use of technology among respondents. In contrast, sex and gender was significantly associated with knowledge of technology after controlling for socio-demographic variables and current technology use. In particular, female respondents were more likely to have some or a great deal of knowledge about technology for caregiving compared to their male counterparts. In addition, the use of technology was also significantly associated with technology knowledge.

**Table 4:** Results of stepwise logistic regression analysis: knowledge of technology (*n* = 299)

Variable	Odds ratio (95% CI)	*p*-value
Knowledge of technology^a^		
Sex and gender (ref: male)	3.93 (1.33–11.63)	0.013
Current use of technology (ref: use)	0.22 (0.086–0.56)	0.0015

Other socio-demographic variables such as age, marital status, ethnicity, education level, rurality, employment status, housing status, family finances, length of care, caregiving relationship, age of care recipient and care recipient’s ADL were not selected for entry into the stepwise regression

^a^Current technology use was also included in the model as a variable

**Table 5:** Results of stepwise logistic regression analysis: use of technology (*n* = 300)

Variable	Odds ratio (95% CI)	*p*-value
Use of technology		
Sex and gender (ref: male)	0.73 (0.27–1.97)	0.54
Age of care recipient	1.05 (1.00–1.10)	0.048

Other socio-demographic variables such as age, marital status, ethnicity, education level, rurality, employment status, housing status, family finances, length of care, caregiving relationship, age of care recipient and care recipient’s ADL were not selected for entry into the stepwise regression

An ordinal logistic regression (Table 6) was conducted to investigate the relationship between caregiver sex and gender and technology costs. Controlling for socio-demographic variables listed in Table 1, female respondents were less willing to pay higher amounts for technology to assist with caregiving compared to their male counterparts. Family finances was then added and found to be significantly associated with technology costs. That is, respondents whose family finances kept them going to the end of the month were more likely to be prepared to pay more for technologies to assist with caregiving than respondents whose family finances were stretched.

**Table 6:** Results of stepwise ordinal logistic regression analysis: cost of technology (*n* = 272)

Variable	Odds ratio (95% CI)	*p*-value
Sex and gender (ref: male)	–	–
$100 - $500 vs < $100	0.60 (0.29–1.22)	0.16
$501 - $1000 vs < $100	0.27 (0.11–0.64)	0.0031
> $1000 vs < $100	0.21 (0.070–0.64)	0.0058
Family finances (ref: does not work out)	–	–
$100 - $500 vs < $100	1.86 (1.08–3.21)	0.026
$501 - $1000 vs < $100	2.74 (1.38–6.05)	0.0013
> $1000 vs < $100	4.79 (1.29–15.85)	0.010

Other socio-demographic variables such as age, marital status, ethnicity, education level, rurality, employment status, housing status, length of care, caregiving relationship, age of care recipient and care recipient’s ADL were not selected for entry into the stepwise regression

To examine the sex and gender influence on potential feature preferences when installing and when using technology, stepwise multinomial regression models (Table 7) were used. While sex and gender was not significantly associated with any feature preferences when controlling for other socio-demographic variables, caregiver education level and family finances were found to be significantly associated with potential feature preferences when first setting up technology after being added to the model. Specifically, respondents with higher education levels (Odds ratio = 0.31, *p* = 0.0005, 95% CI = 0.16, 0.60) and better family finances (Odds ratio = 0.43, *p* = 0.014, 95% CI = 0.22, 0.84) were less likely to pick cost as the most important potential feature when installing technology. The stepwise regression found no additional variables to be significantly associated with feature preferences when either using or installing technology.

**Table 7:** Results of stepwise multinomial regression analysis: feature preferences when setting up technology (*n* = 266)

Variable	Odds ratio (95% CI)	*p*-value
Sex and gender (ref: male)	–	–
Easy to learn to use vs easy to install	0.56 (0.28–1.14)	0.11
Cost vs easy to install	1.69 (0.74–3.86)	0.21
Others vs easy to install	1.06 (0.39–2.89)	0.91
Family finances (ref: does not work out)	–	–
Easy to learn to use vs easy to install	1.18 (0.57–2.45)	0.66
Cost vs easy to install	0.43 (0.22–0.84)	0.014
Others vs easy to install	0.68 (0.28–1.66)	0.39
Education level (ref: high school or less)	–	–
Easy to learn to use vs easy to install	0.79 (0.41–1.55)	0.50
Cost vs easy to install	0.31 (0.16–0.60)	0.0005
Others vs easy to install	0.50 (0.21–1.217)	0.11

Other socio-demographic variables such as age, marital status, ethnicity, rurality, employment status, housing status, length of care, caregiving relationship, age of care recipient and care recipient’s ADL were not selected for entry into the stepwise regression

In summary, male respondents were more willing to pay higher amounts for caregiving technologies while female respondents were more likely to have more knowledge about these technologies. With respect to other socio-demographic variables, caregiver age was significantly associated with perceived usefulness of technology, care recipient age was significantly associated with technology use and family finances were significantly associated with willingness to pay for technologies after controlling for confounders. Finally, feature preferences when installing are mediated by caregiver education level and family finances.

## Discussion

This is one of the first studies, to our knowledge, that examined the needs and preferences for technology across a North American sample of informal caregivers of PWD from a sex and gender perspective. In spite of the documented benefits of technology in caregiving, findings from the survey found more than 80% of respondents have little to no knowledge of technologies that are available to assist with caregiving. Similarly, more than 90% of the respondents had never used technology to help with their caregiving duties. Given the demographic characteristics of the respondents, the findings could be attributed to the caregiver’s age profile. Given that most of the respondents were older adults (> 60 years old), they may not have been exposed to technology designed to assist with caregiving, which corroborated with the narrow range of ADL identified by respondents as areas that technology would assist in. As such, these respondents may be reluctant to adopt new technologies unless they become convinced about the significant benefits conferred by the technology [18, 37]. Hence, future initiatives can explore ways to generate interest among the caregiving population and bridge the gap between the introduction and uptake of technologies designed to assist with caregiving. Given that most current initiatives of technology promotion are in the form of online resource lists and websites from caregiving organizations and support groups across North America [38–40], which may not be accessible to all caregivers, especially those without immediate access to the internet, greater community engagement and education about the benefits of technology use among caregivers are needed. These efforts can include but are not limited to roadshows, demonstrations and fairs during caregiving support groups and events. By demonstrating the usefulness of these devices, caregivers will be better able to appreciate the value that these technologies bring to the caregiving process.

### Knowledge and use of technology

When examining sex and gender influences on the knowledge and use of technology among respondents, sex and gender was found to have a significant association with technology knowledge. Controlling for other socio-demographic variables and technology use, female respondents have significantly more knowledge about technology available for caregiving compared to their male counterparts. Such an association is in contrast with technology perceptions in the general population, which found males reporting more comfort in using computers and have more knowledge about technologies including computers [41, 42]. However, when faced with health related technologies, there has been a general positive attitude and tendency to use the devices regardless of sex and gender [43, 44]. Given the overall lack of knowledge about technologies among respondents and the sex and gender differences, future efforts should focus on effective avenues of informing caregivers about the benefits of technology for caregiving in a manner that takes into account sex and gender distinctions. Specifically, awareness and educational initiatives can be geared more towards male caregivers, who may otherwise not have the opportunity to get in touch with the latest technologies designed for caregiving. While sex and gender were not significantly associated with technology use among respondents, greater care recipient age was found to be associated with greater technology use. As the care recipient ages, their care needs increases. As such, more time and effort is required on the part of the family caregiver to ensure that their loved one remains safe and well. With a heavier caregiving load and burden, family caregivers of older care recipients might be more likely to seek out and adopt technologies that will be able to assist with their caregiving duties [45]. That said, caregiving technologies have been demonstrated to assist caregivers across all levels of burden and these findings represent a first step in identifying specific sub-groups of family caregivers of PWD that may benefit from initiatives to increase technology use and uptake.

### Perceived usefulness of technology

Despite not having much knowledge about existing caregiving technology, respondents were receptive to the use of technologies in assisting them with certain aspects of care. Specifically, they found technology to be useful in having daily conversations with the care recipient, reminding the care recipient to take their medication and of the current time (mean score > 3.5). These findings highlight the aspects of caregiving where respondents would most appreciate assistance and provide a guide for developers when designing new technologies. Specifically, more focus should be directed to creating devices or programs that assist with these care activities.

Technology was not perceived to be useful in assisting with all other ADL of the care recipient including but not limited to preparing food and mobility. Given the lack of technology knowledge and awareness among the respondents, they may not have been familiar with some of the recently introduced technologies such as smart stoves and fall detectors designed to assist with other ADL such as cooking and mobility/fall prevention respectively [11]. As such, respondents may not be in a position to adequately evaluate the usefulness of technology on their caregiving activities as they are not aware of the technologies themselves. This suggests a need for a one-stop comprehensive resource for caregivers to gain an overview of the types and potential benefits of various technologies available to assist with multiple aspects of caregiving. In addition to unlocking the full potential of technology to a wider audience, caregivers would also be able to make more informed judgements about the applicability and usefulness of technology within their own caregiving context.

### Feature preferences of technology

Given the stressful nature of caregiving [46, 47], it is expected that caregivers would prefer technologies with easy installation and operation to avoid bearing additional burden associated with technology set-up. The cost of technology was another concept that emerged at several points throughout the questionnaire. Respondents with higher education levels and better family finances were less likely to select costs as the top feature. Similarly, male respondents and respondents with better family finances were more likely to be willing to pay more for technologies. In line with previous literature involving caregivers of older adults and PWD [11, 48], these findings suggest that caregivers are price-sensitive, which is in-line with the financial demands of caregiving, including transportation, medication support and lost wages due to reduced productivity at work [49]. As expected, respondents with greater financial leverage were more willing to invest in technologies for caregiving. Respondents with higher education levels were also less concerned about the costs of technology. While this relationship could be attributed to the income levels associated with higher education levels, findings from studies examining general technology adoption have suggested that individuals with higher education levels tend to have greater technology knowledge [50], which allows them to better see the value and benefits of these devices in their caregiving routines.

Finally, the sex and gender difference in technology cost is congruent with previous literature on other caregiving populations [48] and can be attributed to the difference in attitudes towards technology. Given the generally more positive attitudes of technology among male respondents [42], they are expected to better recognize the value and purpose of the technology in relation to its cost. By shedding light on the monetary considerations of technology acquisition, these findings highlight the importance for technology developers to factor affordability as an important criterion when bringing technologies to market. Despite certain respondents being willing to pay for technology, government organizations can explore the potential of subsidies and other initiatives to support all caregivers in the adoption of cost-effective, helpful caregiving technologies. Given the costs of long-term care borne by the government every year, substantial savings can be realized by subsidizing caregiving technologies that have demonstrated to delay or even obviate institutionalization [48].

With respect to features when using technology, more than half of the respondents indicated ‘reliability’ as the most important feature, followed by 21.7% respondents who selected the ‘ability for the device to work without manual input’ as the most important feature. In line with the preferences when installing technology, respondents gravitated towards features that allowed them to spend more time on other activities and less on interacting with the technology. In particular, reliable technologies are ones that require less time and fewer resources to respond to issues and breakdowns. Similarly, limiting the manual input required to operate the technology would also contribute to reducing the amount of attention needed from the caregiver and thereby allowing them to focus more on taking care of their family member with dementia. Together, these findings presents a crucial aspect of technology acceptance, specifically a greater acceptability for technologies that were unobtrusive and simple to use [35]. On the contrary, ‘being easy to get help when the technology is broken’ was only identified as the top feature by 11.9% of the respondents. Given that most respondents identified ‘reliability’ as the top feature, respondents may be under the expectation that technology should rarely encounter a breakdown. As such, getting assistance during times of technology breakdown may not be considered by many as a likely scenario. Nonetheless, after-sales support should remain as a priority for technology providers and developers as these experiences can provide feedback on the long-term operation of the devices which would in turn support the refinement and creation of future technology that better align with the preferences of the end users.

### Limitations

The findings present a pioneering overview of the perspectives of these caregivers as it relates to technology use and adoption. One of the main strengths of the study is the questionnaire’s comprehensive coverage of items related to technological needs and preferences including the use, knowledge, features and connection with the care recipient’s ADL. As such, it ensured a more comprehensive understanding of respondents’ perspectives on this area of growing importance. However, this study was also subject to several limitations. One potential shortcoming of this study is the amount of missing data. In addition to the exclusion of 52 responses due to missing socio-demographic information, responses were missing for each of the dependent variables and excluded from the analyses. As such, cautionary interpretation of the survey responses is warranted.

Given the significant number of non-English speaking informal caregivers residing in Canada, the language criteria and unavailability of the questionnaire in other languages may have excluded many of these potential respondents. As such, future work should include multilingual study materials and utilize random respondent selection in order to obtain a more representative sample of the caregiving population.

While the offering of a self-administered survey provided respondents with greater convenience, it may have resulted in non-response bias due to the low response rates. It is acknowledged that most respondents in the survey were females and a direct measure of gender such as the Masculine Gender Role Stress [51] and Bem-Sex-Role-Inventory [52] was not applied in this survey. These gender measures are important as they help to shift the conceptualization of gender away from the binary and enable a better understanding of how different facets of gender affect technology perceptions among caregivers. Given the nature of the work, the analyses were limited to the items included in the original questionnaire, which may not reflect the current conceptualizations of sex and gender. Specifically, while the questionnaire item asked respondents for their gender, the response options were sex-based terms (i.e. male and female). As understanding the impact of the multiple aspects of sex and gender on technology perceptions is becoming increasingly important, future work in this area should attempt to include representative samples of caregivers based on sex and gender as well as consider the incorporation of a direct gender measure or the development of a gender index based on pre-collected variables [53].

Finally, given the nature of the secondary analysis, we were unable to capture in-depth perspectives of caregivers as it relates to technology and incorporate additional control variables not collected in the original survey. Therefore, future work can further this area of research through theoretically informed qualitative research involving caregivers in order to gather a deeper understanding of their technology perceptions as well as the impact of other factors such as social exclusion, care recipient’s level of dementia severity and prior training on their needs and preferences of technology.

## Conclusion

As one of the first studies that examines the technology needs and preferences of family caregivers of PWD from a sex and gender lens, the findings have significant policy and practice implications. Specifically, more community engagement initiatives and incentive programs are warranted to enhance the awareness and uptake of technologies respectively. Additionally, significant sex and gender differences between male and female caregivers with respect to the knowledge of technology were uncovered through the survey. Given the paucity of information on the sex and gender differences in caregiving as well as an increased focus on sex and gender initiatives around the world, these findings represent a pioneering step towards the incorporation of sex and gender influences in technology design and promotion for caregivers. Nonetheless, future efforts are warranted to build on the current study and establish an in-depth understanding of these sex and gender influences as well as other social and environmental factors through qualitative methodologies such as interviews and focus groups.

## Supplementary information

**Additional file 1.**

## Data Availability

The datasets generated and analysed during the current study are not publicly available as the ethics approval that was obtained only allowed participant data to be shared among the project researchers and stored in a secure place within the research premises.

## Abbreviations

ADL: Activities of Daily Living
GPS: Global Positioning System
PWD: Persons with Dementia
SD: Standard Deviation
TAM: Technology Acceptance Model
