# Supplementary material for: Sex and gender differences in technology needs and preferences among informal caregivers of persons with dementia
Source: BMC Geriatr. 2020 May 18;20:176. doi: 10.1186/s12877-020-01548-1 (PMC7236350; doi:10.1186/s12877-020-01548-1)
Supplement: Supplementary file 1 — Additional file 1. [file 12877_2020_1548_MOESM1_ESM.docx]

Supplementary File 1. Questionnaire items used in the analyses

**Technology Knowledge**

How much do you know about Intelligent Assistive Technology that supports cognitive disabilities like dementia?

A great deal

Some

Not much

Nothing at all

**Technology Use**

Have you ever used Intelligent Assistive Technology to help care for a person with dementia?

Yes

No

**Perceived usefulness of technology to assist in care recipient’s Activities of daily living**

Intelligent Assistive Technology may be helpful to people with dementia and their caregivers when they are completing various tasks and /or activities. The following is a list of activities of daily living. Using the scale from 0 to 5, with “0” indicating “Would not benefit at all” from the use of an intelligent assistive technology, and “5” indicating “Would benefit a great deal” from the use of an intelligent assistive technology, please indicate how much you think your Care Recipient would benefit from having help with the activity.

| Activities of daily living | How much would assistance from an Intelligent Assistive Technology device help the Care Recipient? | | | | | |
| --- | --- | --- | --- | --- | --- | --- |
| 1. Preparing food | 0 | 1 | 2 | 3 | 4 | 5 |
| 2. Eating | 0 | 1 | 2 | 3 | 4 | 5 |
| 3. Preparing a drink (e.g. coffee or tea) | 0 | 1 | 2 | 3 | 4 | 5 |
| 4. Drinking | 0 | 1 | 2 | 3 | 4 | 5 |
| 5. Getting dressed | 0 | 1 | 2 | 3 | 4 | 5 |
| 6. Taking a shower | 0 | 1 | 2 | 3 | 4 | 5 |
| 7. Brushing teeth | 0 | 1 | 2 | 3 | 4 | 5 |
| 8. Taking a bath | 0 | 1 | 2 | 3 | 4 | 5 |
| 9. Using the toilet | 0 | 1 | 2 | 3 | 4 | 5 |
| 10. Mobility | 0 | 1 | 2 | 3 | 4 | 5 |
| 11. Reminding the Care Recipient of the time | 0 | 1 | 2 | 3 | 4 | 5 |
| 12. Reminding the Care Recipient of the location | 0 | 1 | 2 | 3 | 4 | 5 |
| 13. Having daily conversation with the Care Recipient when no one’s around | 0 | 1 | 2 | 3 | 4 | 5 |
| 14. Making phone calls | 0 | 1 | 2 | 3 | 4 | 5 |
| 15. Reminding the Care Recipient to do the housework | 0 | 1 | 2 | 3 | 4 | 5 |
| 16. Reminding the Care Recipient to take their medication | 0 | 1 | 2 | 3 | 4 | 5 |
| 17. Paying bills | 0 | 1 | 2 | 3 | 4 | 5 |
| 18. Washing hands | 0 | 1 | 2 | 3 | 4 | 5 |

**Feature preferences when installing technology**

The following is a list of 6 potential features of Intelligent Assistive Technology that would be relevant when first setting up the device. Please rank them in order of importance by placing the numbers 1 through 6, with “1” representing the most important feature and “6” the least important, in the space provided beside each feature. Only use each number once.

___ Easy to install

___ Clear operating instructions

___ Easy to learn how to use

___ Availability of training

___ Aesthetics of the technology

___ Cost

**Feature preferences when using technology**

The following is a list of 6 potential features of Intelligent Assistive Technology that would be relevant when using the device. Please rank each potential feature in order of importance with “1” representing the most important feature and “6” representing the least important. Only use each number once.

___ Easy to get help if device is broken

___ Reliability of device

___ Ability for the system to work without manual input from the user

___ Ability to set-up features on the device and customize its operation

___ Ability to receive performance reports about user performance and the system operation

___ Accessible outside of the home (e.g. via Internet, smart phone, etc.)

**Technology costs**

If you could buy an Intelligent Assistive Technology Device that can help you with providing care to your Care Recipient, how much would you be willing to pay for it?

Less than $100

$100 - $500

$501 - $1000

Over $1000
